# *Mycobacterium tuberculosis* ribosomal protein S1 (RpsA) and variants with truncated C-terminal end show absence of interaction with pyrazinoic acid

**DOI:** 10.1038/s41598-020-65173-z

**Published:** 2020-05-20

**Authors:** Katherine Vallejos-Sánchez, Juan M. Lopez, Ricardo Antiparra, Emily Toscano, Harry Saavedra, Daniela E. Kirwan, L. M. Amzel, R. H. Gilman, Helena Maruenda, Patricia Sheen, Mirko Zimic

**Affiliations:** 1 Laboratorio de Bioinformática, Biología Molecular y Desarrollos Tecnológicos. Laboratorios de Investigación y Desarrollo. Facultad de Ciencias y Filosofía. Universidad Peruana Cayetano Heredia, Lima, Perú; 2 Pontificia Universidad Católica del Perú, Departamento de Ciencias, Sección Química, Centro de Espectroscopía de Resonancia Magnética Nuclear (CERMN), Lima, Perú; 3 Department of Biophysics and Biophysical Chemistry, Johns Hopkins University School of Medicine, Baltimore, MD. USA; 4 Infection and Immunity Research Institute, St George’s, University of London, London, England; 5 International Health Department. Johns Hopkins School of Public Health, Baltimore, MD USA

**Keywords:** Biophysics, Molecular biology

## Abstract

Pyrazinamide (PZA) is an antibiotic used in first- and second-line tuberculosis treatment regimens. Approximately 50% of multidrug-resistant tuberculosis and over 90% of extensively drug-resistant tuberculosis strains are also PZA resistant. Despite the key role played by PZA, its mechanisms of action are not yet fully understood. It has been postulated that pyrazinoic acid (POA), the hydrolyzed product of PZA, could inhibit *trans*-translation by binding to Ribosomal protein S1 (RpsA) and competing with tmRNA, the natural cofactor of RpsA. Subsequent data, however, indicate that these early findings resulted from experimental artifact. Hence, in this study we assess the capacity of POA to compete with tmRNA for RpsA. We evaluated RpsA wild type (WT), RpsA ∆A438, and RpsA ∆A438 variants with truncations towards the carboxy terminal end. Interactions were measured using Nuclear Magnetic Resonance spectroscopy (NMR), Isothermal Titration Calorimetry (ITC), Microscale Thermophoresis (MST), and Electrophoretic Mobility Shift Assay (EMSA). We found no measurable binding between POA and RpsA (WT or variants). This suggests that RpsA may not be involved in the mechanism of action of PZA in *Mycobacterium tuberculosis*, as previously thought. Interactions observed between tmRNA and RpsA WT, RpsA ∆A438, and each of the truncated variants of RpsA ∆A438, are reported.

## Introduction

Tuberculosis (TB), caused by *Mycobacterium tuberculosis* (MTB), is responsible for 1.4 million deaths and 10.4 million new cases of disease each year^1^.

Depending on various factors, including the immune response of the host^2^, MTB may either cause latent infection or active disease. It is estimated that latent infection affects one quarter of the population worldwide who do not have clinical manifestations of the disease but have an estimated 2.4–10% lifetime risk of activation and development of active TB^3,4^.

Pyrazinamide (PZA) is an anti-tuberculosis antibiotic used in first- and second-line regimens. It reduces the treatment period from nine to six months and diminishes the risk of relapse due to its activity against semi-dormant mycobacteria^5^. PZA is a pro-drug that enters the bacteria by passive diffusion and hydrolyzes to its active form, pyrazinoic acid (POA)^6^.

The POA is then expelled out of the mycobacterium by an efflux mechanism. If the pH of the extracellular environment is acidic, the POA is protonated to POAH, re-enters the cell, and the proton is released in the cytosol. This cycle repeats itself, causing an intra-cellular accumulation of POA and a reduction in the pH of the cytoplasm. This leads to a lethal alteration of membrane permeability; however, the exact mechanism by which this occurs is not yet known^7^ It is important to remark, that other recent studies, found that extracellular acid pH is not required to have PZA/POA achieve its lethal effect^8–10^. This confirms that the real mechanism of action of PZA is actually more complicated than though, and significant further research is required.

It has been demonstrated that the anti-bacterial properties of PZA could be associated with various mechanisms of action, including the alteration of membrane energy production^7^, inhibition of *trans*-translation^11,12^, inhibition of pantothenate and CoA biosynthesis^13^, and inhibition of Fatty Acid synthetase (FAS I)^14^.

RpsA is a ribosomal protein that has been extensively studied in *Escherichia coli (E. coli)*. Its main functions include: participation in the initiation of translation^15,16^; the recognition of messenger RNA (mRNA) bearing weak or degenerated Shine-Dalgarno sequences^17,18^; and participation in *trans*-translation^19^.

During protein biosynthesis the ribosome can be stalled by an mRNA which has lost its stop codon, by otherwise defective mRNA, or in the absence of tRNA during translation^20^. *Trans*-translation is a system in which transfer-messenger RNA (tmRNA), which is encoded by the *ssrA* gene, interacts with Small protein B (SmpB)^21^, elongation factor Tu (EF-Tu)^22^, and RpsA^19,23^ to form a ribonucleoprotein complex that efficiently releases the stalled ribosome.

In mycobacteria, studies investigating *trans*-translation have shown that exposure to ribosome-targeting antibiotics results in the upregulation of tmRNA expression, although the mechanism through which this takes place remains unknown^22^. It has been suggested that POA interacts with RpsA, and by doing so inhibit *trans*-translation, with lethal consequences for metabolically dormant mycobacteria^11^. In contrast, Personne and Parish^24^ claim that PZA may not directly affect *trans*-translation in MTB, because SmpB deletions and *ssrA* gene mutations did not generate noticeable changes to the bacterial growth pattern. Furthermore, Yang *et al*.^12^ crystallized the fourth domain of MTB RpsA with and without POA. The residues Phe307, Phe310, His322 and Arg357 were found to interact with POA, as well as with tmRNA^12^. PZA-resistant clinical strain, DHM 444, was reported^11^ to harbor an alanine deletion at position 438 (∆Ala438) on the unstructured carboxy terminal (C-terminal) end of RpsA. It was suggested that this deletion blocked the interaction between POA and RpsA, preventing the lethal inhibition of *trans*-translation and resulting in resistance to PZA^11^.

In contrast to these findings, Dillon *et al*., using Isothermal Titration Calorimetry found no evidence of an interaction between RpsA and POA^25^. This study applied allelic exchange to PZA-susceptible laboratory MTB strain H37Ra. No significant differences were found in the PZA MICs in RpsA ∆Ala438 when compared to the wild type strain, and there was no inhibition of *trans*-translation by POA. The authors also showed that neither ∆Ala438 nor RpsA over-expression conferred resistance to PZA, in contrast to previous reports^11,26^.

Based on these discrepancies, the present study evaluated the interaction between RpsA and POA, as well as between RpsA and tmRNA, for both the RpsA wild type (RpsA WT) and RpsA ∆A438 proteins, using a range of techniques including Electrophoretic Mobility Shift Assay (EMSA), Isothermal Titration Calorimetry (ITC), Microscale Themophoresis (MST) and Nuclear Magnetic Resonance spectroscopy (NMR).

**Figure 1:**
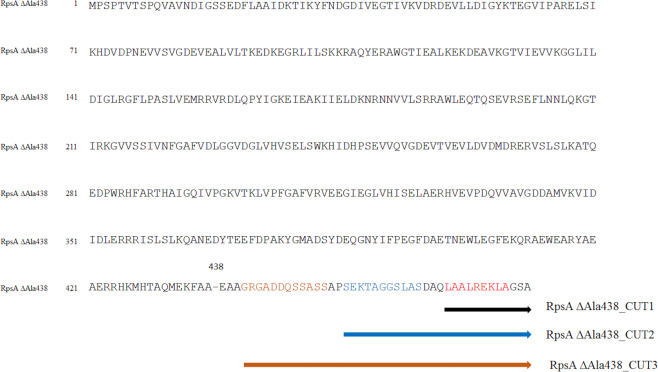
Cutting scheme for the generation of truncated proteins at the C-terminal end of RpsA ∆A438.

Considering that MTB RpsA is composed of four S1 domains and a highly flexible C-terminal end^27,28^, two hypotheses were addressed in order to understand why ∆A438 deletion in RpsA protein induced resistance^11^. The first hypothesis proposed that the ∆Ala438 mutation favors movement of the non-structured C-terminal end of MTB RpsA, leading to blockage, by steric impediment, of the entry of POA to the MTB RpsA binding site. The second hypothesis postulates that the ∆Ala438 mutation induces a conformational change within the POA binding site, reducing its overall affinity. To evaluate these hypotheses, RpsA variants with truncated C-terminal regions were carefully designed (RpsA ∆Ala438_CUT1, RpsA ∆Ala438_CUT2, and RpsA ∆Ala438_CUT3) to limit the capacity of the non-structured end to block the POA binding site. Thereby enable POA to recover its ability to bind to MTB RpsA, assuming that this was lost as a result of the ∆Ala438 mutation Fig. 1.

## Results

### Production of RpsA

RpsA and all its variants were purified as soluble proteins. In all cases, native 5% PAGE and ethidium bromide stained revealed the presence of nucleic acids bound to the purified proteins. Treatment with DNase and RNase showed that these nucleic acids were RNA (Supplementary Figure S1).

### Electrophoretic mobility shift assay (EMSA) to test the interaction between tmRNA, RpsA WT and RpsA ∆A438

**Figure 2:**
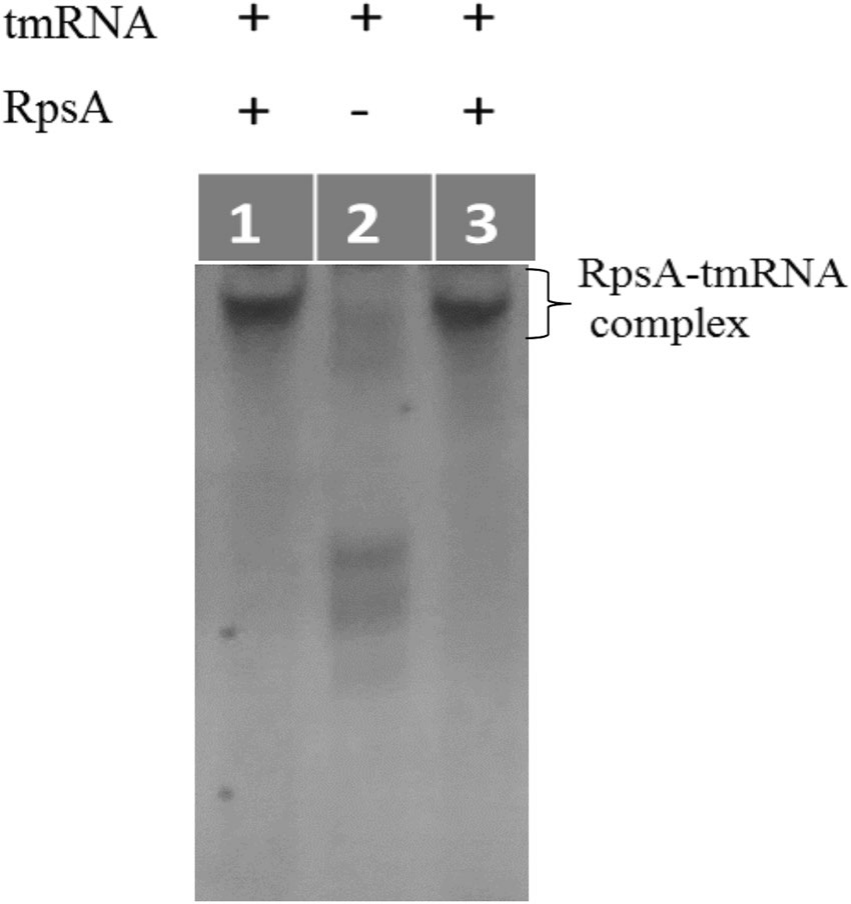
Electrophoretic Mobility Shift Assay (EMSA) to evaluate interactions between tmRNA and RpsA WT and RpsA ∆Ala438. Lane 1: RpsA WT and tmRNA; Lane 2: tmRNA; Lane 3: RpsA ∆A438 and tmRNA.

EMSA is a simple technique based on the identification of the electrophoretic mobility pattern of the nucleic acid when it is free and when it forms complexes with the target protein, as in the latter case migration will usually be slower^29^. Free tmRNA showed greater electrophoretic mobility than tmRNA incubated in the presence of RpsA WT or RpsA ∆A438 (Fig. 2), confirming an interaction between both RpsA variants with tmRNA, as described previously^11,12^.

### Microscale thermophoresis (MST) to test the interaction between tmRNA and RpsA WT

**Figure 3:**
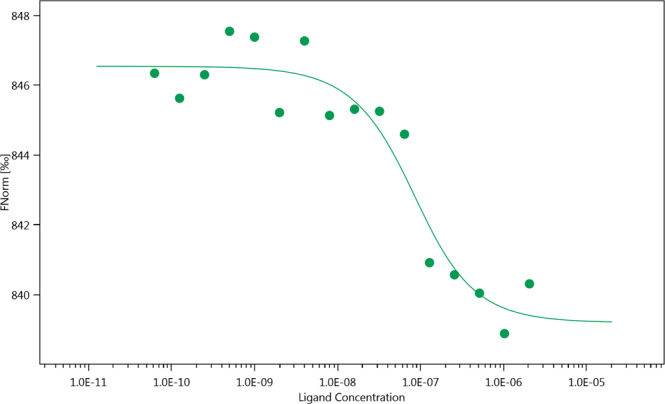
Evaluation of the interaction between RpsA WT (Red-tris-NTA) and tmRNA by Microscale Thermophoresis (MST). Kd= 58.1 +/− 2.95 nM. Monolith NT.115 peak.

MST is a relatively new approach that requires few sample quantity, fast, easy and the only requirement for its use was to bind covalently the protein with a fluorophore^30^. Microscale thermophoresis was used to confirm the interaction between RpsA WT and tmRNA, and to determine the dissociation constant (Fig. 3). Its variability was calculated after data fitting and estimation of parameters, yielding a K_d_ value of 58.1 +/− 2.95 nM.

### Electrophoretic mobility shift assay (EMSA) to test the interaction between POA and RpsA variants

**Figure 4:**
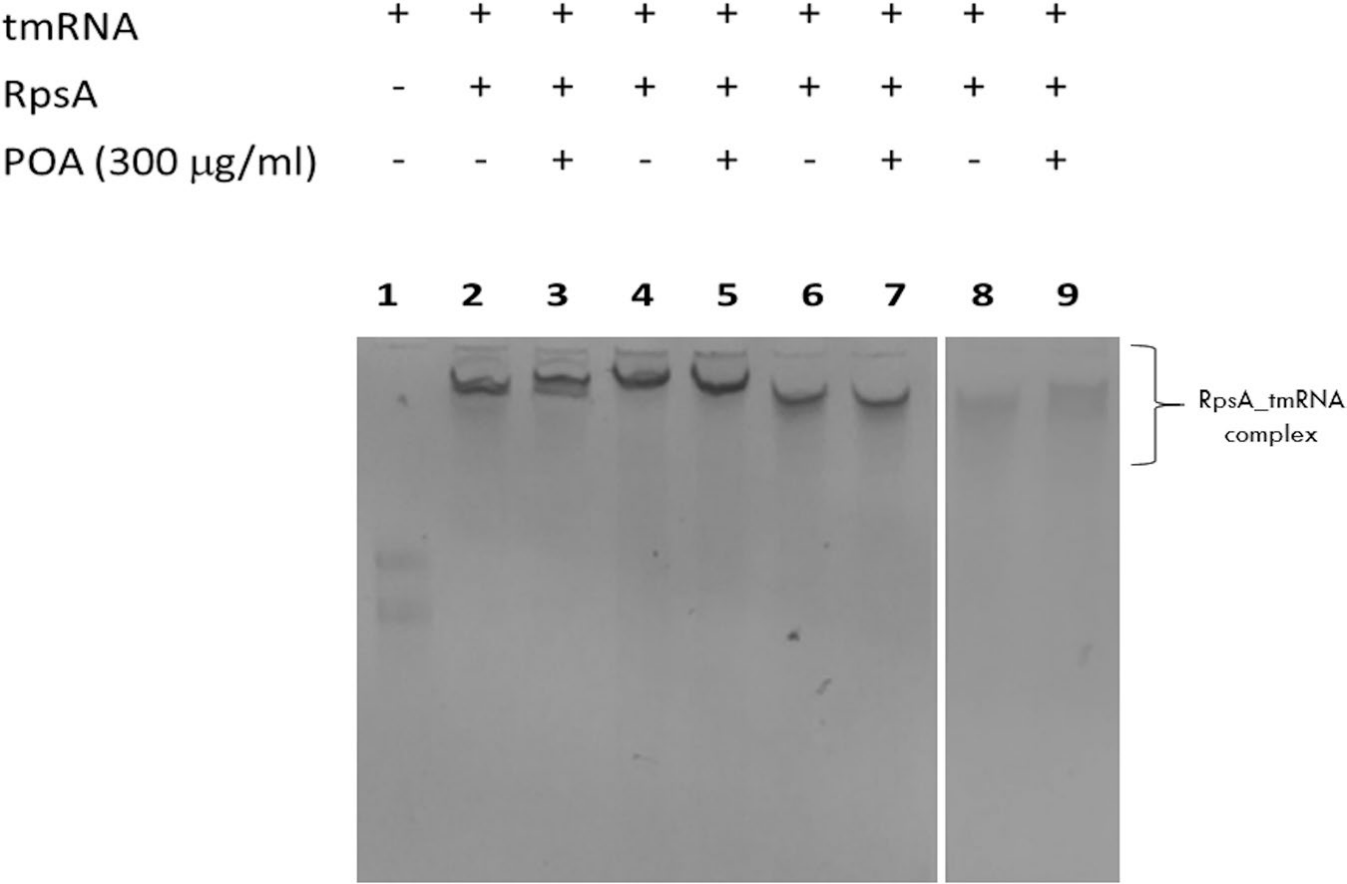
Competition between tmRNA and POA for RpsA (EMSA). Lane 1: tmRNA; Lane 2–3: RpsA WT; Lane 4–5: RpsA ∆A438; Lane 6–7: RpsA ∆A438_CUT1: Lane 8–9: RpsA ∆A438 _CUT3. Two gels are shown with equal running conditions (gel 1: Lanes 1–7; gel 2: Lanes 8–9).

tmRNA was able to positively bind to all RpsA variants (Fig. 4, lanes 2, 4, 6 and 8). However, displacement of tmRNA at saturating concentrations of POA was not observed for any of the protein variants tested (RpsA WT, RpsA ∆Ala438, and the truncated variants, Fig. 4, lanes 3, 5, 7 and 9), indicating that POA does not compete with tmRNA for RpsA.

### Isothermal titration calorimetry (ITC) to test the interaction between POA and RpsA variants

**Figure 5:**
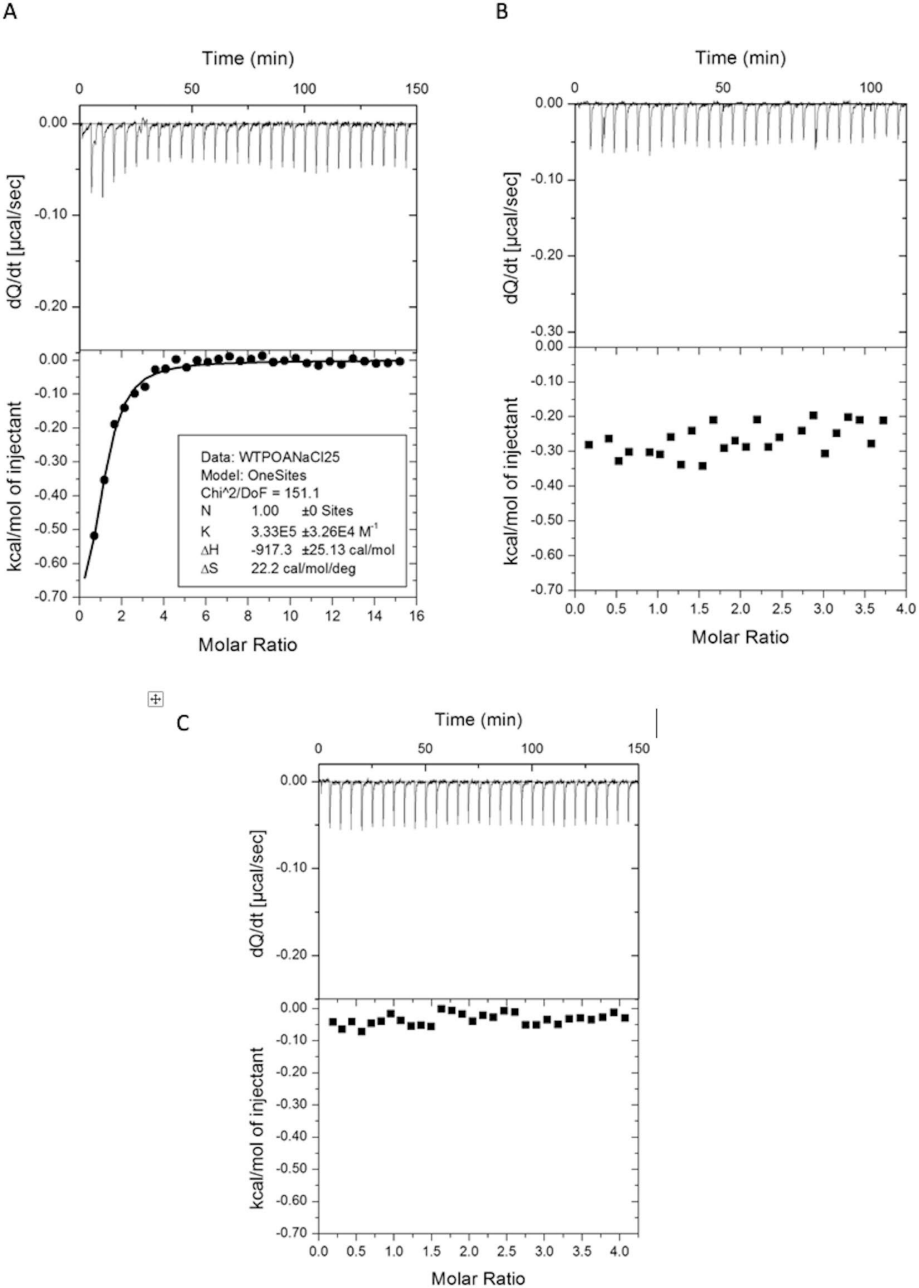
Evaluation of the interaction between RpsA and RpsA ∆A438 and POA, determined by Isothermal Titration Calorimetry (ITC). (**A,B**) RpsA WT, without (**A**) and with (**B**) pH correction and (**C**) RpsA ∆A438.

ITC is a technique to determine the thermodynamic parameters from drug binding interactions with proteins in their native state, without chemical modification or adhesion to any surface^31,32^. We found no variation in thermodynamic parameters during ITC. Highly acidic POA generated a dilution heat signal when the pH of the assay was not conditioned to overcome the pH change induced by POA (Fig. 5A). When adjusted, the results clearly indicate no affinity between RpsA and POA for either RpsA WT, RpsA ∆A438 (Fig. 5B, 5C), or the truncated variants RpsA ∆Ala438_CUT1, RpsA ∆Ala438_CUT2, and RpsA ∆Ala438_CUT3 (Supplementary Figure S2).

### Nuclear magnetic resonance spectroscopy (NMR) to test the interaction between POA and RpsA variants

NMR is a powerful tool used to determine molecular interactions^33^. One of the simplest methods to investigate protein–ligand interactions by NMR is to observe the Chemical Shift Perturbation (CSP) induced by complex formation. If the interactions are in the nanomolar or sub-nanomolar affinity range, the resonances are usually observed in the slow NMR exchange regime in which two signals can be observed, one coming from the free ligand and the other from the ligand complex. In the case of a micromolar range affinity protein-ligand interaction, the signals involved are usually detected by the fast NMR exchange regime in which the two signals collapse into one, and the total displacement of the chemical shift depends on the equilibrium concentrations between the free and the bound state.

**Figure 6:**
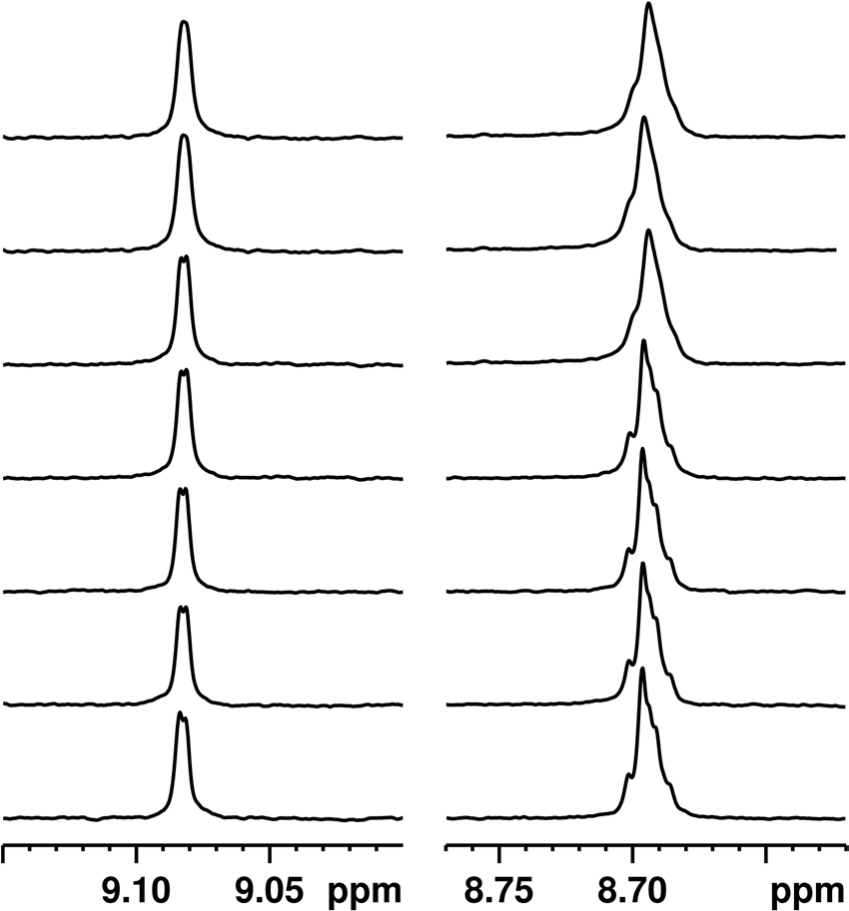
Chemical Shift Pertubation (CSP) experiment: ^1^H NMR spectrum of 100 μM POA with different concentrations of RpsA. From bottom to top: 0 μM, 6.6 μM, 13.1 μM, 26.3 μM, 52.5 μM, 157.5 μM, and 315 μM RpsA.

The NMR spectrum of POA is composed of downfield aromatic signals (1H_ortho_: 9.07 ppm, 1H_meta_ and 1H_para_: 8.7 ppm), none of which overlap with signals associated with the protein (Supplementary Figure S3). Fig. 6 shows the signals generated by POA against increasing concentrations of RpsA WT. No evidence of any CSP or integral diminishment of POA NMR signals was observed, even in the presence of more than three times an excess of RpsA WT. However, at high RpsA WT concentrations a small change in the line-width of POA signals is observed, indicative of a change in relaxation.

To confirm these results, we measured the diffusion coefficient of POA in the absence and presence of RpsA WT (7.1 × 10^−10^m^2^/s and 7.0 × 10^−10^m^2^/s, respectively; supplementary Figure S4). In the bound state, the ligand is expected to acquire the diffusion coefficient of the protein (1.4 × 10^−10^ m^2^/s), which would result in a lower apparent diffusion rate, depending on the average time spent in the bound and unbound state^33,34^. There was no reduction in the apparent diffusion coefficient, even in the presence of excess RpsA WT (supplementary Figure S4).

**Figure 7:**
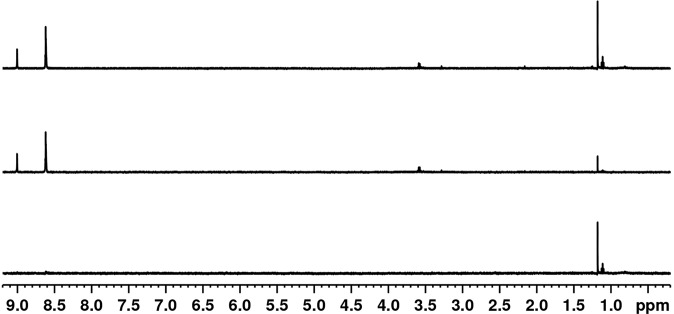
Saturation transfer difference (STD) experiment using 200 μM POA and 4.5 μM RpsA: (top) Off resonance spectrum; (middle) On resonance spectrum; (bottom) STD spectrum.

**Figure 8:**
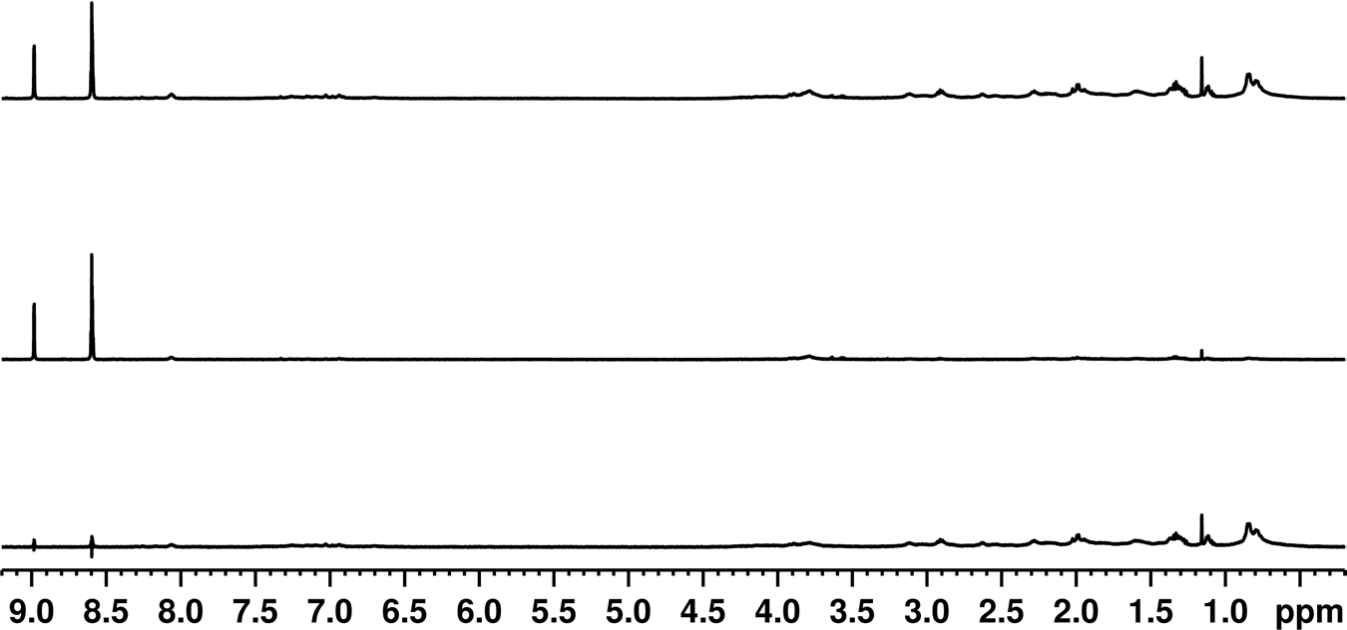
Saturation transfer difference (STD) experiment using 500 μM POA and 50 μM RpsA: (top) Off resonance spectrum; (middle) On resonance spectrum; (bottom) STD spectrum.

To evaluate the possibility of an affinity at the millimolar level, we performed Saturation Transfer Difference (STD) experiments^35^. This method is based on the selective saturation of the protein signals which are transferred to the ligand by spin diffusion. This method allows the determination of affinities in the micro- and millimolar range. First, we performed a series of STD experiments at low RpsA WT concentrations (1.1 μM, 3 μM and 4.5 μM). No saturation transfer between RpsA WT and POA was detected (Fig. 7, Supplementary Figure S5 and S6). Next, high concentration (50 μM RpsA WT) STD experiments with different RpsA/POA molar ratios (1/2, 1/5, 1/7, 1/10 and 1/20) were tested. Only marginal saturation transfer was observed (Fig. 8 and Supplementary Figures S8, S9, S10 and S11). This small STD signal could be explained by a weak interaction (in the mM range), which is too low to be detected under the conditions tested (Supplementary Figure S12). In any case, this weak interaction, even high concentrations of POA, is not sufficient to explain the competitive effect between POA and tmRNA claimed in earlier studies^11^.

## Discussion

Shi *et al*.^11^ suggested that PZA may be inhibiting *trans*-translation or the salvage of ribosomes by dissociating the tmRNA-RpsA complex. In the same year, Andini and Nash^22^ postulated that the *trans*-translation mechanism may be vital for the survival of mycobacteria. In the study by Shi *et al*. (2011), the ∆Ala438 deletion located at the highly flexible C-terminal end of the MTB RpsA protein in a PZA-resistant clinical strain was reported, and it was suggested that this mutation may have been responsible for the change to a PZA resistant phenotype. Based on this finding, in the present study we sought to investigate and understand the effect of the ∆Ala438 deletion on the conserved RpsA protein^36^ and on its relationship with the mechanism of resistance to PZA.

Andini and Nash^22^ previously reported that *Mycobacterium smegmatis*, a naturally PZA resistant mycobacteria, has between 500 and 1000 copies of tmRNA per cell, a ratio of 1:10 or 1:20 tmRNA:ribosomes, similar to *E. coli*^37^. Assuming that this ratio is similar in MTB, according to the hypothesis presented by Shi *et al*. (2011) we would expect that POA would compete with tmRNA, saturating the sites of interaction between tmRNA and ribosomes, either by a high intracellular POA concentration or by having an affinity greater than that of tmRNA.

To evaluate the postulated POA/RpsA interaction and the ability of POA to displace tmRNA from RpsA we employed a range of experimental approaches, as well as different C-terminal truncated variants of RpsA. Our results consistently showed that POA does not compete with tmRNA for RpsA, since no displacement of tmRNA from RpsA or any of its variants was observed in the presence of excess POA. This finding contrasts with what has previously been reported^11,12^.

We found that purified MTB RpsA protein recombinant expressed in *E. coli* was bound to RNA (Supplementary Figure S1). This has also been previously observed with *E. coli* RpsA^38^. Although we took steps to eliminate RNA from our purified recombinant RpsA, this was not performed in the studies conducted by Shi *et al*. (2011) or by Yang *et al*. (2015). This omission may have led to background noise in the interaction assays performed in those studies, and the subsequent mis-interpretation of this noise as a positive finding. Having controlled for RNA contamination in the purified RpsA, we consider that our results are free of any potential confounding caused by it. We believe future studies are required to evaluate the consequences of potential contamination with RNA of purified RpsA from *M. tuberculosis* and other mycobacterias as *M. smegmatis*.

In addition, EMSA analysis showed that both the RpsA WT and RpsA ∆Ala438 proteins were able to form complexes with tmRNA. Fan *et al*.^39^ approximated the binding between RpsA and tmRNA by generating a truncated RpsA protein comprising residues 280–438 (only the fourth domain and the C-terminal end), and evaluated its interaction with two variants of the mRNA domain of MTB tmRNA. The binding was estimated to have an affinity constant between 23.56 +/− 5.74 and 92.17 +/− 17.80 × 10^4^ M^−1^ ^39^, which partially characterizes the affinity between RpsA and tmRNA. In our study, the apparent binding constant between RpsA WT (full length protein and truncated variants) and MTB tmRNA was estimated at 1.72 +/− 0.873 × 10^7^ M^−1^, compared to the 1 × 10^8^ M^−1^ previously reported for *E. coli* tmRNA^40^. Therefore, the positive control (RpsA-tmRNA) used in our interaction model is both qualitatively and quantitatively consistent with published data.

As previously reported by Dillon^25^, ITC measurements of RpsA WT and POA interactions generated interesting results. The addition of POA (pK_a_ 2.9) without pH adjustment to the protein solution led to an ITC signal corresponding to − 917.3 +/− 25.13 cal/mol (Fig. 5A), a value that could be misinterpreted as a molecular interaction. However, considering that the energy of a hydrogen bond varies from 1–40 Kcal/mol^41,42^, the thermodynamic parameters are unlikely to correspond to a specific bond between these molecules but rather to a dilution heat differential. When the pH was corrected (Fig. 5B), this phenomenon was no longer present and no interaction between RpsA WT and POA was observed. To further confirm the type of interaction between RpsA and POA we made use of several NMR techniques CSP, DOSY and STD being able to determine affinity from the order of nano, micro and millimolar, respectively, as previously demonstrated in previous studies evaluating interactions between PZA, POA and a variety of PZA analogs with fatty synthase I (FAS I) protein^43^. The NMR results showed that POA has a weak interaction for RpsA (in the mM range), an affinity that was unable to compete against the strong binding to tmRNA, even at high POA concentrations.

The X-ray crystal structure of RpsA bound to POA, showing the interaction between POA and the RpsA S1 domain, has been published^12^. The S1 domain was depicted using NMR spectroscopy 1H-15N HSQC^12^. However, experimental evaluation of the ligand-protein interaction was not shown. Moreover, in a subsequent paper, Fan and coauthors studied the interaction of the RpsA S1 domain with tmRNA using NMR^39^ and, surprisingly, the tmRNA-POA competition experiment was not addressed. The results we present here show low affinity of POA for RpsA at a level (mM) at which it is difficult to compete with the strong binding reported between RpsA and tmRNA (nM)^11,39^. The weak POA-RpsA interaction observed here by STD suggests that POA has co-crystallized with RpsA, possibly through a crystallization artifact generated by the high concentration crystallization conditions^12^.

Our experimental results are robust and consistent with each other across the different techniques used. They contrast with the results reported by Shi *et al*. (2011) and by Yang *et al*. (2015), which described a strong interaction between RpsA WT and POA with an affinity constant of 7.53 × 10^6^ M^−1^ and 3.56 × 10^5^ M^−1^, respectively. Our data also confirm the findings obtained by Dillon *et al*. (2017) who also found no evidence of an interaction between RpsA and POA using ITC^25^.

Finally, in recent years there has been an attempt to relate mutations in RpsA to PZA resistance, based on the approaches discussed above. The sequencing of this gene has demonstrated mutations in both PZA-sensitive and -resistant strains. However, of the large number of RpsA mutations reported in strains susceptible to PZA (Q162R, D37A, K45E, L53V, A112T; I26L, I26V, E67V, G214V, N221H; K45Q, E197K, V254G, D268N, E405K, M432T) and resistant strains (R474W, R474L, E433D, K45N, I101T, Y164C, ∆Ala437, L138R, S151P, K167N, L185P, INSERT C193, E201G, R212W, K213Q, G214S, G229A, H244Q, R289L, S324P, V338A, G341S, INSERT G341, A376E, A412V, E413Q, D446A, S450T; R212R, M432T, F310F; K45E; V260I; DEL I26, V74I, K93R, L206L, K277R, S324F, E325K, G341R, D342N, A344P, I351F, T370P, W403G)^44–49^, very few have been found to be consistently associated with resistance to PZA. Of note, strain DHM444^50^ with mutation ∆Ala438 is the only clinical strain reported to harbor this mutation on RpsA. This strain was described like “less susceptible to PZA, with a borderline or low-level resistance (MIC, 200 to 300 mg/mL, which is two to three times the MIC used to define susceptible strains)”^51^. Shi *et al*. generated the mutations ∆Ala438 and D123A in the MTB chromosome and confirmed PZA resistance (300 μg/mL)^52^.

It is important to emphasize that potential protein-protein interactions taking place *in vivo* between RpsA, SmpB, tmRNA and other compounds of trans-translation machinery may offer an explanation for the contradictory results reported so far. More studies are needed to establish a model to determine how RpsA mutations are associated with resistance to PZA, as this relationship remains unclear^53,54^.

In conclusion, our experimental results present evidence that there is no interaction between RpsA WT and POA. We also show that the binding between MTB RpsA WT and tmRNA is not displaced by POA under the conditions applied in the present study. These findings suggest that RpsA may not be involved in the mechanism of action of PZA in MTB, as was previously thought.

## Materials and Methods

### Cloning, expression, and purification of RpsA

The *rpsA* wild type (*rpsA* WT) gene was amplified by PCR from MTB H37Rv strain DNA. Proteinase K digestion^55^, digestion with BamHI and XhoI, and ligation into a pET28a plasmid (Novagen) with an N-terminal His-tag and Kanamycin resistance gene were performed.

MTB *rpsA* ∆A438 complete gene, *rpsA* ∆A438_CUT1 (468 aa), *rpsA* ∆A438_CUT2 (454 aa), and *rpsA* ∆A438_CUT3 (440 aa) (Fig. 1) were synthesized and cloned in a pET28a vector (Genemed Synthesis, Inc).

All recombinant proteins were expressed in *E. coli* BL21 (DE3). The cells were grown at 37 °C to an optical density (O.D) of 0.6–0.8, and expression was induced with 0.5 mM IPTG at 25 °C for 8 hours. The bacteria were centrifuged (15 min at 4 °C and 6 000 rpm) and the pellet resuspended in 20 mM Tris-HCl buffer, pH 8, 300 mM NaCl. Cells were disrupted by three freeze-thaw cycles, followed by three sonication cycles on ice (1 min active, 1 min inactive). Debris was separated by centrifugation (13 000 rpm x 15 min), and the supernatant was recovered by a further centrifugation step (13 000 rpm x 15 min). Samples were treated with DNase and RNase to determine the type of nucleic acid associated with the purified protein. Treated samples were migrated in native PAGE on twin gels: one was stained with Coomasie blue and the other with ethidium bromide. Finally, all samples were processed with RNase A for 16 hours at 4 °C.

Purification was achieved by Ni^2+^ affinity chromatography^56^, using 10 mM to 500 mM Imidazole (linear gradient). The purified fractions were concentrated by ultrafiltration using Amicon (Millipore) 10 KDa cutoff filters; they were washed repeatedly with phosphate-buffered saline (PBS) until all the imidazole had been removed. Protein concentration was determined by measuring absorbance at 280 nm (extinction coefficient: ε = 46410 M^−1^ cm^−1^, estimated by the ExPASy ProtParam tool (http://web.expasy.org/protparam/))^57^. Additionally, in order to evaluate the presence of any remaining nucleic acids in the purified proteins, absorbance at 260 nm was measured, and twin gels (5% native polyacrylamide) were processed and stained with ethidium bromide and Coomassie blue.

### Production of tmRNA

The *ssrA* gene was amplified from 10 ng MTB H37Rv genomic DNA using primers at 0.5 μM which included the T7 promoter sequence: Forward 5′-TAA TAC GAC TCA TCA TAG GAT CTG ACC GGG AAG TTA ATG GC-3′ and reverse 5′-GAT CAG ATC CGG ACG ATC GGC ATC G-3′^11^. Phusion flash PCR Master mix (Thermo scientific F-548L) was used with a final volume of 20 μL. Cycling parameters included 98 °C for 10 sec, 70 °C for 10 sec and 72 °C for 15 sec, ending with a cycle of 72 °C for 1 min. The 620 bp products were purified using the High Pure Clean-Up kit following the manufacturer’s instructions (Roche-life technologies).

From the amplified and purified product, *in vitro* transcription was performed using the TranscriptAid T7 High Yield Transcription Kit as previously described^11^. Briefly, the transcription of 1 μg of DNA was carried out at 37 °C for 2 hours. Purification of the 602 bp product was performed using the Thermo scientific geneJET RNA purification kit, ranging from 2–6 μg/μL.

### Electrophoretic Mobility Shift Assay (EMSA) to test the interaction between tmRNA and RpsA WT

tmRNA (10 pmol) was prepared as previously described^11^. RpsA WT (10 μM), 5% glycerol, 80 mM KCl, 9 mM MgCl_2_, 100 μg/mL BSA, and 20 U RNasin were combined in a final volume of 20 μL and incubated at room temperature for 1 hour. Samples were mixed with 5 μL REMSA loading buffer 5× (Thermo Scientific) and analyzed by electrophoresis (5 mA for 4 hours) in 5% polyacrylamide gel at 0.5X TBE, then stained with ethidium bromide.

### Microscale Thermophoresis (MST) to test the interaction between tmRNA and RpsA WT

RpsA was labeled using the MO-L008 Monolith His-tag Labeling kit RED-tris-NTA following the protocol available from the manufacturer (100 nM final concentration in PBS-T buffer). A 16-step dilution series was undertaken by adding 10 μL MST buffer with 0.1% Tween 20 to 15 tubes. Twenty μL of 4.1 μM tmRNA was placed in the first tube, and 10 μL was transferred to the second tube and mixed well by pipetting. This was repeated to generate a 1:1 dilution series. 10 μL of RpsA was added to each tube (final concentration 50 nM). The samples were incubated at room temperature for 15 minutes then loaded into Standard Monolith NT capillaries. The capillaries were scanned using a Monolith NT.115 MST instrument (medium MST power, 5% excitation power) and the data analyzed using MO Affinity Analysis software v2.2.4 (NanoTemper Technologies GmbH). The measured fluorescence was normalized and averaged, and the binding constant was determined using a global fit calculation and a 1:1 fitting model. A rough estimate of the affinity constant with its 95% confidence interval was obtained after fitting the experimental data with the theoretical corresponding function (NanoTemper Technologies Software Analysis).

### Electrophoretic Mobility Shift Assay (EMSA) to test the interaction between POA and RpsA variants

The interaction between POA with RpsA WT, RpsA ∆A438, and the truncated variants of RpsA ∆A438 was indirectly evaluated by measuring a change in the electrophoretic mobility of the tmRNA when bound to RpsA and the displacement of tmRNA after adding POA. The methodology was similar to that described above. POA (300 μg/mL) was used for the competition study with tmRNA. Under these conditions, the POA:tmRNA and POA:RpsA molar ratios were 5:1 and 25:1 respectively.

### Isothermal Titration Calorimetry (ITC) to test the interaction between POA and RpsA variants

Binding affinity was measured at 25 °C by Isothermal Titration Calorimetry (ITC) using a VP-ITC instrument (Microcal Inc, Northhampton, MA). RpsA proteins (20 μM) were dialyzed and prepared in ITC buffer consisting of 10 mM phosphate buffer (Na_2_HPO_4_ – NaH_2_PO_4_), pH 6.0, and 150 mM NaCl. Protein concentrations were determined with a UV spectrometer using an extinction coefficient (ε) of 46410 M^−1^ cm^−1^. POA (550 μM, FLUKA Chemika) samples were prepared in the same ITC buffer, and ligand concentrations were determined by weight per volume. Ligand was injected 30 times (10 μL per injection during 12 s) into the cell containing the protein solution at intervals of 300 s. The stirring speed was 300 rpm. Feedback Mode/Gain was high and filter period was 2 s.

Binding parameters were determined from isotherms after subtracting the average heat of dilution (~ −0.2 kcal/mol) using the MicroCal Data Analysis software (Malvern, Westborough, MA). Experiments were conducted in duplicate for each protein.

### Nuclear Magnetic Resonance spectroscopy (NMR) to test the interaction between POA and RpsA variants

POA stock solution (10 mM) was dissolved in 100 mM phosphate buffer containing150 mM NaCl. After carefully adjusting the pH to 6.4 it was lyophilized and resuspended in D_2_O. RpsA samples were buffer-exchanged with D_2_O buffer containing 100 mM phosphate, pH 6.4 and 150 mM NaCl using Zeba Spin Desalting Columns (7 K MWCO, 2 mL).

All NMR experiments were recorded at 25 °C on a Bruker Avance III HD 500 MHz spectrometer equipped with SMART probe. 1H-NMR experiments were acquired using the Watergate suppression technique to eliminate residual solvent signal, with 384 scans, 64k data points, a spectral width of 10 kHz, and a total relaxation time of 6.28 s.

Diffusion Ordered Spectroscopy (DOSY) experiments were recorded using Watergate pulsed-field gradient stimulated echo (PFGSE) pulse sequence with a 500 ms diffusion delay, 300 µs bipolar gradients, gradient strengths of 2.17, 6.50, 10.84, 15.17, 19.50, 23.84, 28.17, 32.51, 36.84, 41.17 gauss/cm, 256 scans per gradient strength, 64k data points, a spectral width of 10 kHz, and a total relaxation time of 13.28 s. Translational diffusion coefficients were determined using a Stejskal–Tanner equation^58^.

Saturation Transfer Difference (STD) experiments were acquired using Watergate suppression. Selective protein saturation was achieved by applying a series of 1 ms low power (0.019727 Watts) Gaussian shaped pulses and a total saturation time of 6 s. ‘On’ resonance frequency was set at 0.84 ppm, and ‘off’ resonance frequency at 100 ppm^35^.

The flip angle was calibrated for each individual sample using 360° pulse optimization. Spectra were Fourier transformed with 0.3 Hz exponential apodization, and phase and baseline corrected using Topspin 3.5 pl5 software.

## Supplementary information

Supplementary Information.
