# Supplementary material for: Mycobacterium tuberculosis ribosomal protein S1 (RpsA) and variants with truncated C-terminal end show absence of interaction with pyrazinoic acid
Source: Sci Rep. 2020 May 20;10:8356. doi: 10.1038/s41598-020-65173-z (PMC7239899; doi:10.1038/s41598-020-65173-z)
Supplement: Supplementary file 1 — Supplementary Information. [file 41598_2020_65173_MOESM1_ESM.pdf]

Mycobacterium tuberculosis ribosomal protein S1 (RpsA) and variants with truncated C-terminal end show absence of interaction with pyrazinoic acid

Vallejos-Sánchez Katherine[1], Lopez Juan M.[2], Antiparra Ricardo[1], Toscano Emily[1], Saavedra Harry[3], Kirwan Daniela E.[4], Amzel LM[3], Gilman Robert H.[5], Maruenda Helena[2], Sheen Patricia[1], Zimic Mirko[1*]

[1] Laboratorio de Bioinformática, Biología Molecular y Desarrollos Tecnológicos. Laboratorios de Investigación y Desarrollo. Facultad de Ciencias y Filosofía. Universidad Peruana Cayetano Heredia, Lima, Perú.

[2] Pontificia Universidad Católica del Perú, Departamento de Ciencias, Sección Química, Centro de Espectroscopía de Resonancia Magnética Nuclear (CERMN), Lima, Perú.

[3] Department of Biophysics and Biophysical Chemistry, Johns Hopkins University School of Medicine, Baltimore, MD. USA.

[4] Infection and Immunity Research Institute, St George's, University of London.

[5] International Health Department. Johns Hopkins School of Public Health. Baltimore, MD. USA

*corresponding author

E-mail: mirko.zimic@upch.pe

SUPPLEMENTARY DATA

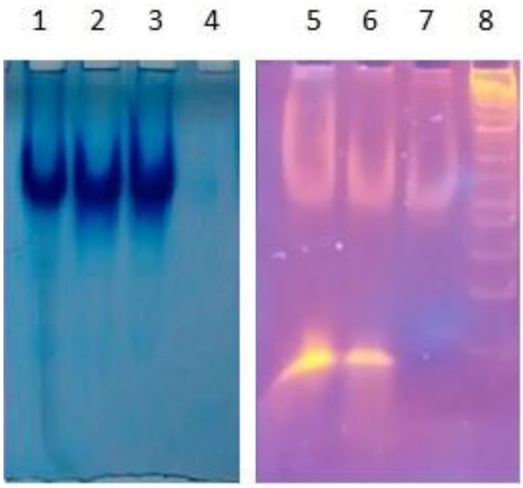

Figure S1. Native polyacrylamide gels identical to 5%, left staining with Coomasie blue and right staining with Ethidium bromide. Lane 1 and 5 RpsA purified by affinity chromatography, Lane 2 and 6 RpsA purified by affinity chromatography incubated with DNase, Lane 3 and 7 RpsA purified by affinity chromatography incubated with RNase. Lane 4 and 8 Riboruler RNA Marker.

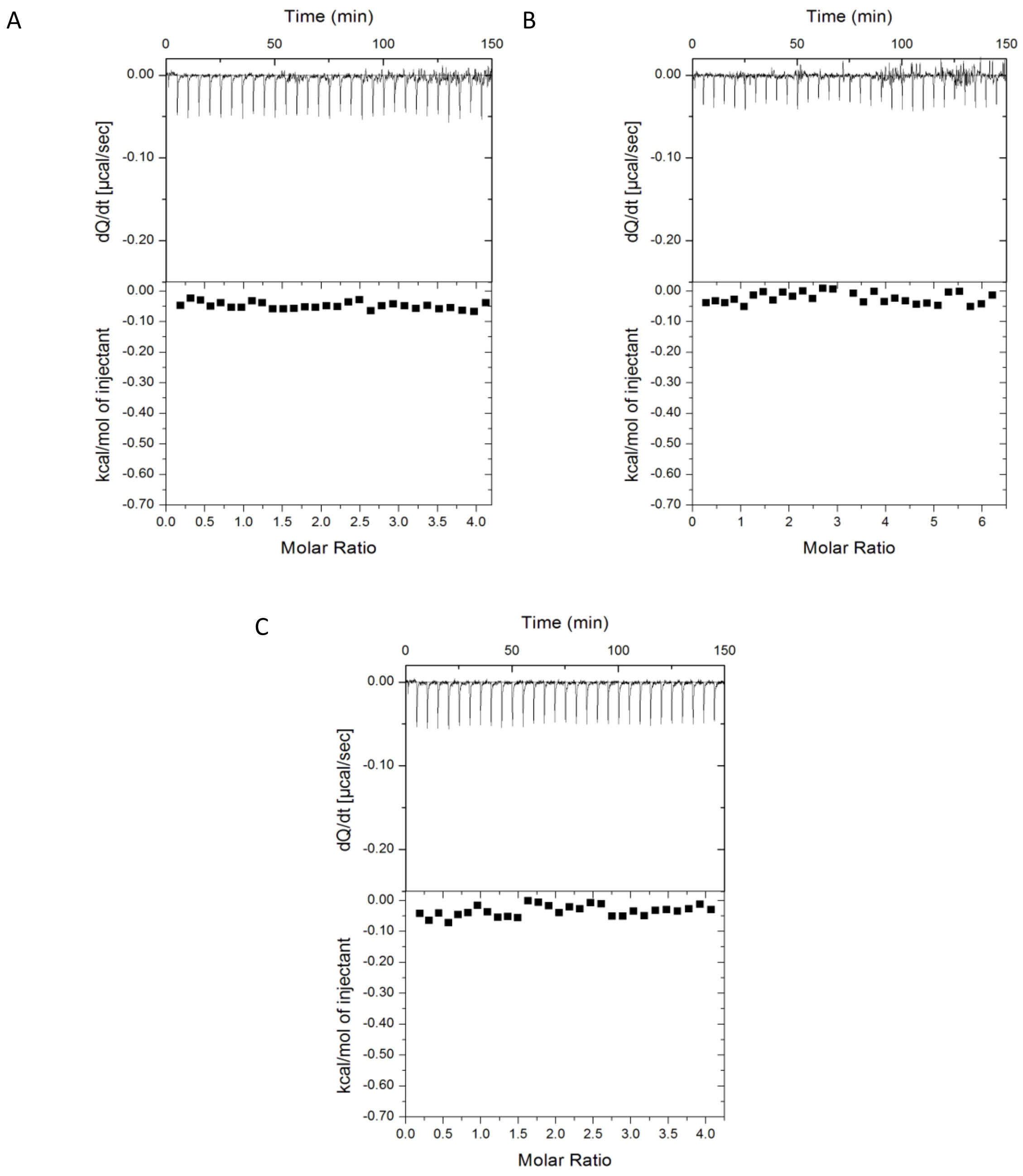

Figure S2. Evaluation of the interaction between RpsA ∆A438 truncated variants and POA, determined by Isothermal Titration Calorimetry (ITC). A) RpsA∆A438_ CUT1. B) RpsA∆A438_CUT2, C) RpsA ∆A438_CUT3.

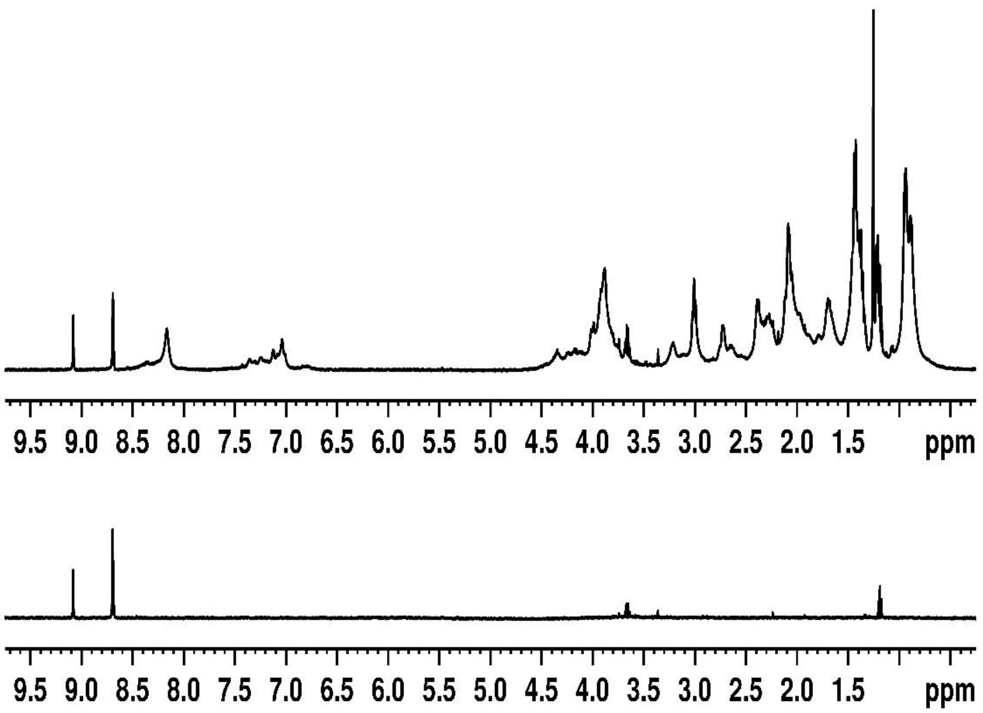

Figure S3. (top) ^{1}H NMR spectrum of 100 μM POA with 325 μM RpsA. (bottom) ^{1}H NMR spectrum of 100 μM POA without RpsA.

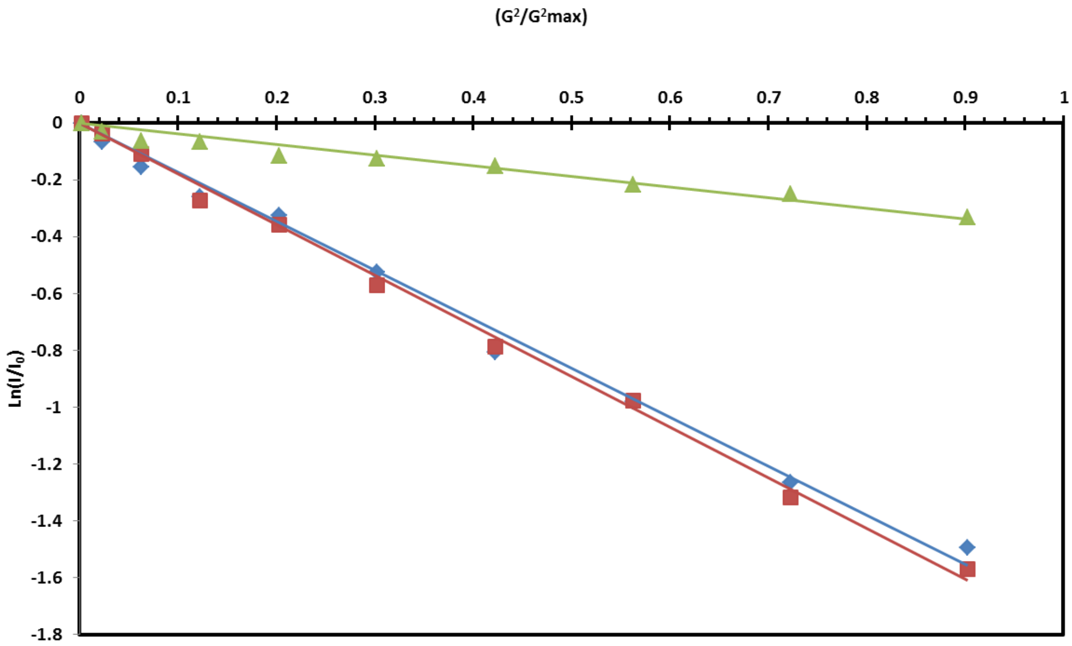

Figure S4: the graph shows the variation of the signal intensity as a function of the gradient strength using the linearized Stejskal–Tanner equation. in bleu diamonds 100 µM POA signal (8.7 ppm) without RpsA, in red rectangles and green triangles POA (8.7 ppm) and RpsA (0.8 ppm) signals respectively in a mixture of 100 POA with 325uM RpsA.

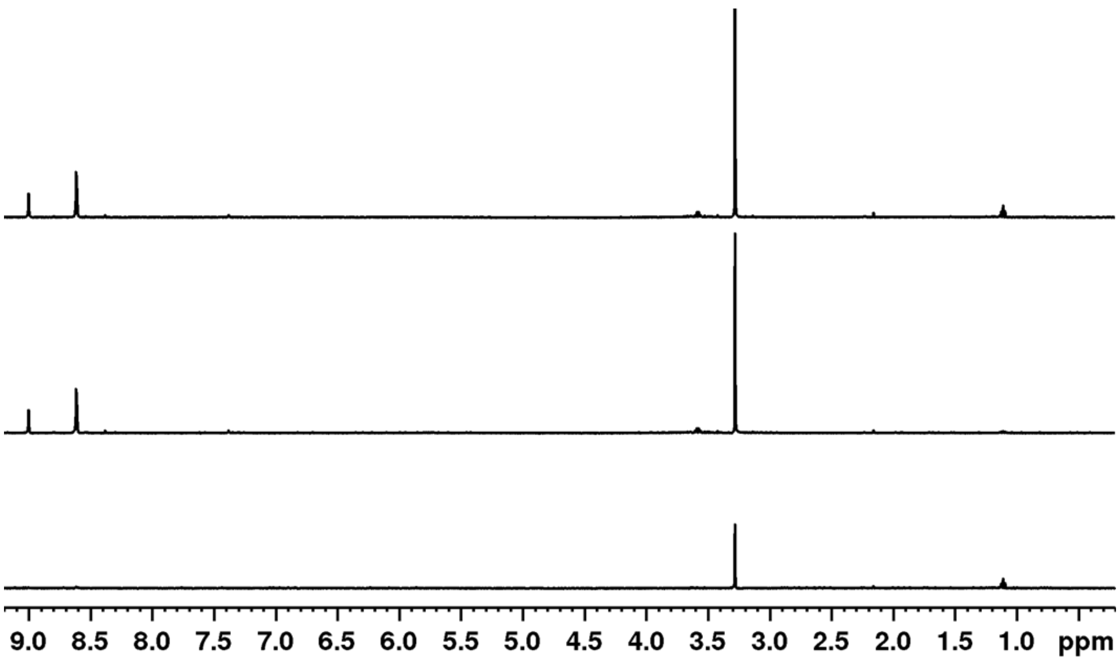

Figure S5. STD control experiment: (top) Off resonance spectrum of 100 µM POA without RpsA. (middle) On resonance spectrum of 100 µM POA without RpsA. (bottom) STD spectrum of 100 µM POA without RpsA.

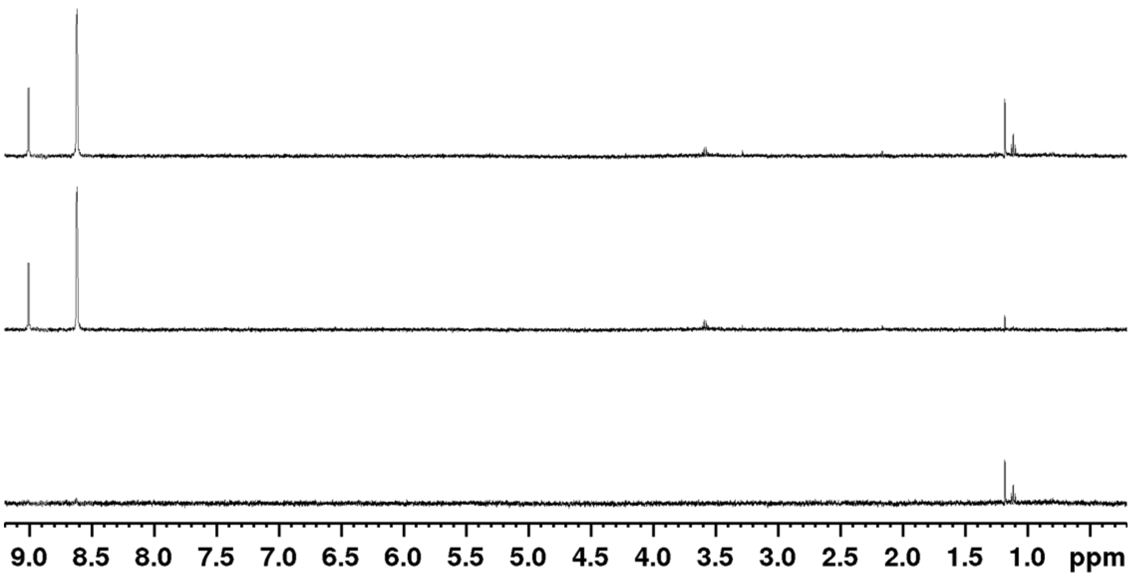

Figure S6. STD experiment: (top) Off resonance spectrum of 200 µM POA with 1.1 µM RpsA. (middle) On resonance spectrum of 200 µM POA with 1.1 µM RpsA. (bottom) STD spectrum of 200 µM POA with 1.1 µM RpsA.

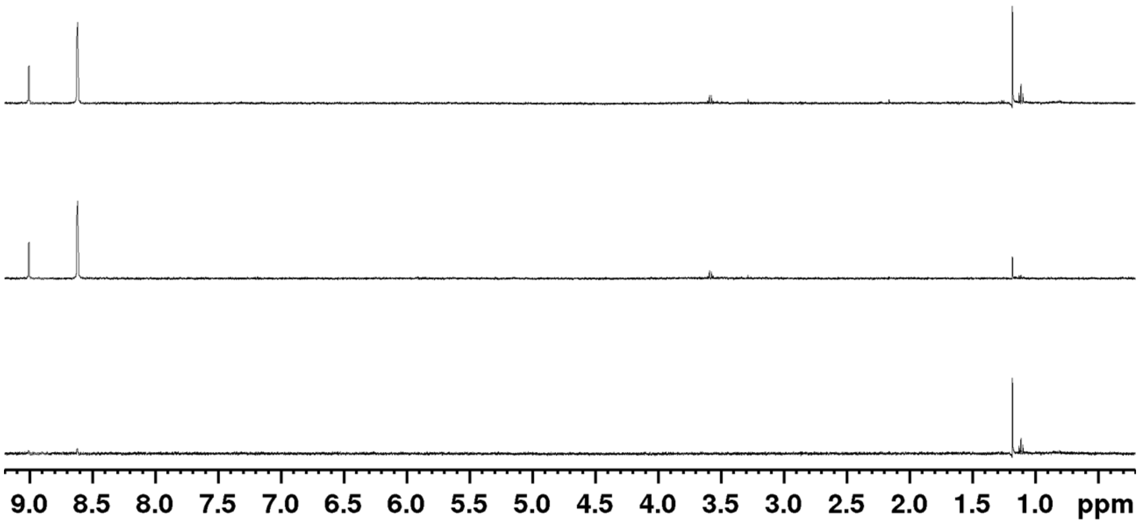

Figure S7. STD experiment: (top) Off resonance spectrum of 200 μM POA with 3 μM RpsA. (middle) On resonance spectrum of 200 μM POA with 3 μM RpsA. (bottom) STD spectrum of 200 μM POA with 3 μM RpsA.

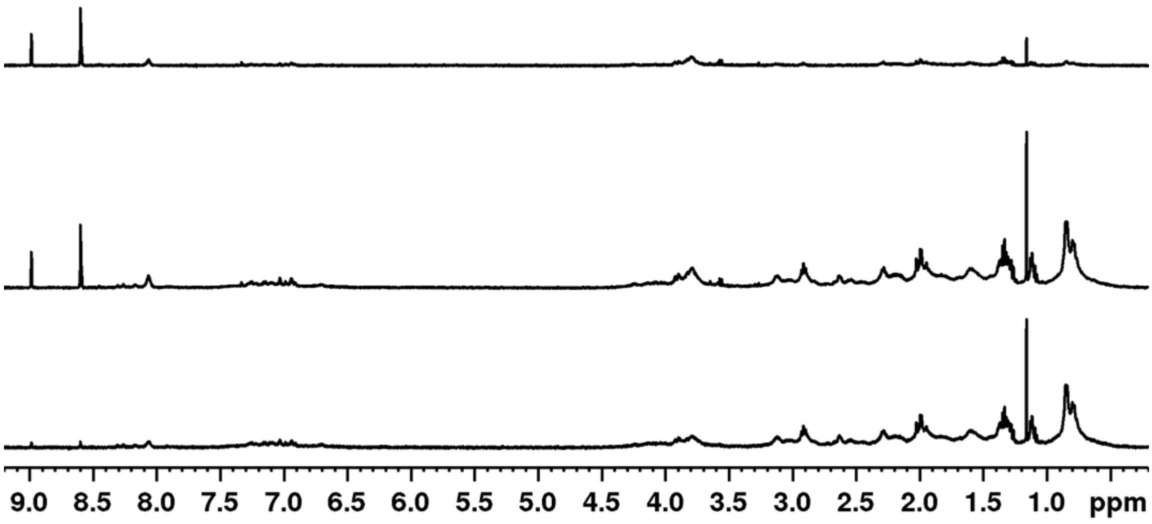

Figure S8. STD experiment: (top) Off resonance spectrum of 100 μM POA with 50 μM RpsA. (middle) On resonance spectrum of 100 μM POA with 50 μM RpsA. (bottom) STD spectrum of 100 μM POA with 50 μM RpsA.

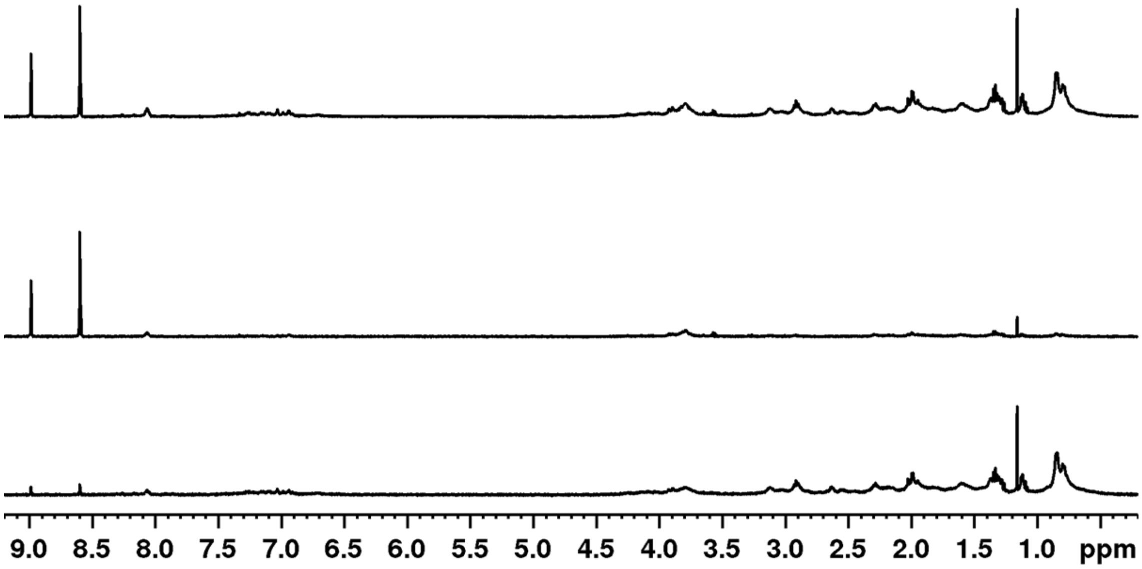

Figure S9. STD experiment: (top) Off resonance spectrum of 250 μM POA with 50 μM RpsA. (middle) On resonance spectrum of 250 μM POA with 50 μM RpsA. (bottom) STD spectrum of 250 μM POA with 50 μM RpsA.

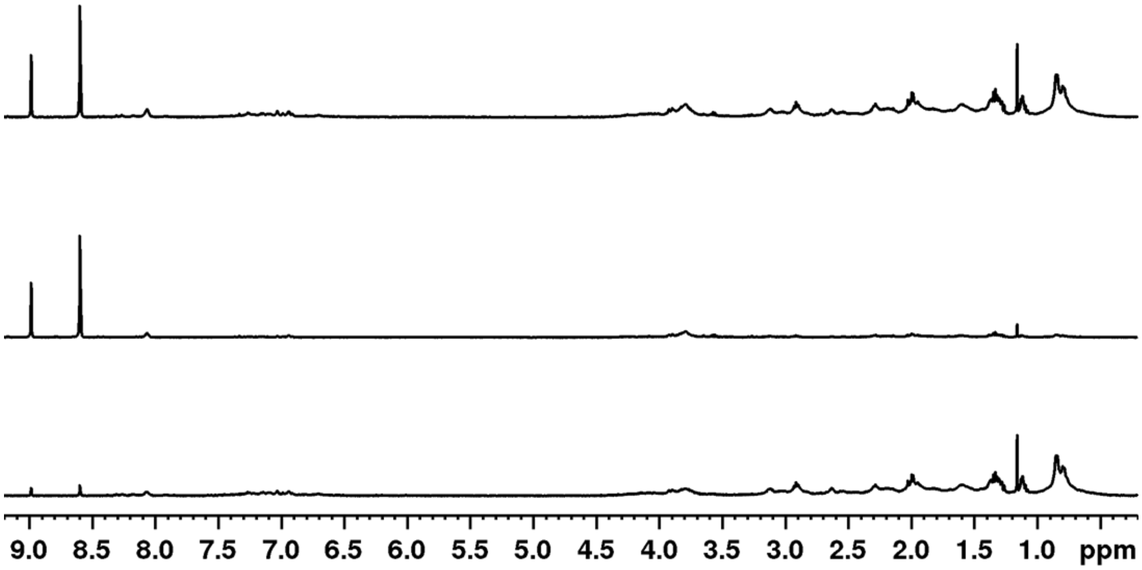

Figure S10. STD experiment: (top) Off resonance spectrum of 350 μM POA with 50 μM RpsA. (middle) On resonance spectrum of 350 μM POA with 50 μM RpsA. (bottom) STD spectrum of 350 μM POA with 50 μM RpsA.

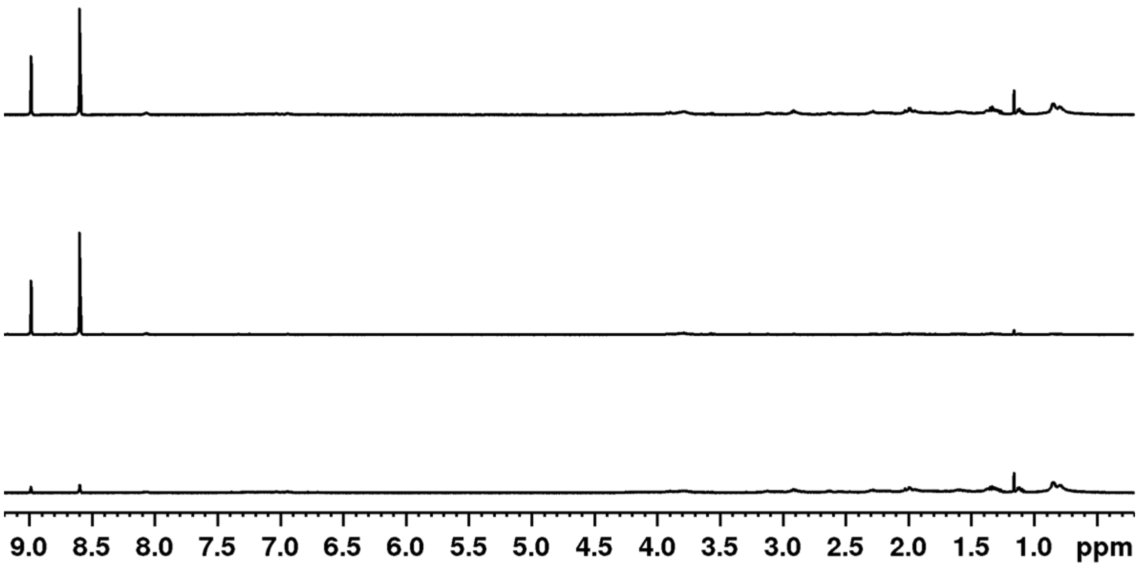

Figure S11. STD experiment: (top) Off resonance spectrum of 1000 μM POA with 50 μM RpsA. (middle) On resonance spectrum of 1000 μM POA with 50 μM RpsA. (bottom) STD spectrum of 1000 μM POA with 50 μM RpsA.

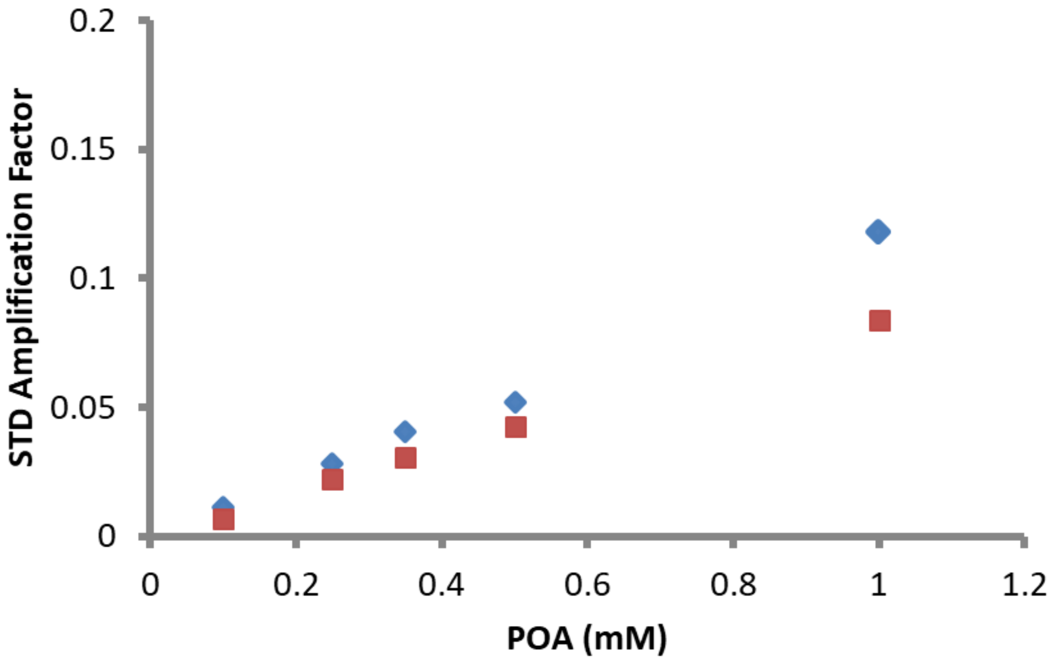

Figure S12. Variation of STD Amplification Factor with POA concentration (blue diamonds 8.7 ppm signal and red rectangles 9.07 ppm signal). The concentration of RpsA was fixit to 50 μM. The dissociation constant could not be determined since in these conditions the amplification factor does not reach its maximum plateau value.
